# Novel MRI-guided focussed ultrasound stimulated microbubble radiation enhancement treatment for breast cancer

**DOI:** 10.1038/s41598-023-40551-5

**Published:** 2023-08-21

**Authors:** Archya Dasgupta, Murtuza Saifuddin, Evan McNabb, Ling Ho, Lin Lu, Danny Vesprini, Irene Karam, Hany Soliman, Edward Chow, Sonal Gandhi, Maureen Trudeau, William Tran, Belinda Curpen, Greg Stanisz, Arjun Sahgal, Michael Kolios, Gregory J. Czarnota

**Affiliations:** 1 Department of Radiation Oncology, Sunnybrook Health Sciences Centre, 2075 Bayview Avenue, T2, Toronto, ON M4N3M5 Canada; 2 Department of Radiation Oncology, University of Toronto, Toronto, Canada; 3 Physical Sciences, Sunnybrook Research Institute, Toronto, Canada; 4 Department of Medical Oncology, Sunnybrook Health Sciences Centre, Toronto, Canada; 5 Department of Medicine, University of Toronto, Toronto, Canada; 6 Department of Medical Imaging, Sunnybrook Health Sciences, Toronto, Canada; 7 Department of Medical Imaging, University of Toronto, Toronto, Canada; 8 Department of Biophysics, University of Toronto, Toronto, Canada; 9 Canada Research Chair in Cancer Imaging, Canadian Institutes of Health Research, Toronto, Canada; 10 Ryerson University, Toronto, Canada

**Keywords:** Cancer, Breast cancer, Cancer therapy

## Abstract

Preclinical studies have demonstrated focused ultrasound (FUS) stimulated microbubble (MB) rupture leads to the activation of acid sphingomyelinase-ceramide pathway in the endothelial cells. When radiotherapy (RT) is delivered concurrently with FUS-MB, apoptotic pathway leads to increased cell death resulting in potent radiosensitization. Here we report the first human trial of using magnetic resonance imaging (MRI) guided FUS-MB treatment in the treatment of breast malignancies. In the phase 1 prospective interventional study, patients with breast cancer were treated with fractionated RT (5 or 10 fractions) to the disease involving breast or chest wall. FUS-MB treatment was delivered before 1st and 5th fractions of RT (within 1 h). Eight patients with 9 tumours were treated. All 7 evaluable patients with at least 3 months follow-up treated for 8 tumours had a complete response in the treated site. The maximum acute toxicity observed was grade 2 dermatitis in 1 site, and grade 1 in 8 treated sites, at one month post RT, which recovered at 3 months. No RT-related late effect or FUS-MB related toxicity was noted. This study demonstrated safety of combined FUS-MB and RT treatment. Promising response rates suggest potential strong radiosensitization effects of the investigational modality.

**Trial registration:** clinicaltrials.gov, identifier NCT04431674.

## Introduction

Radiation therapy (RT) forms an essential component in the management of various malignancies and is utilized in approximately 40–60% of patients in their oncological management^1,2^. RT can be used as a treatment modality in the adjuvant setting (for residual and microscopic disease) or neoadjuvant strategy (for disease downstaging or improving resectability), or for cure (for gross unresectable disease). Radiation also has a vital role in palliative management in advanced or metastatic disease for symptom alleviation. In the past few decades, tremendous technological advances have been achieved in the delivery of RT using image-guided radiotherapy, intensity-modulated radiotherapy, magnetic resonance imaging-linear accelerator (MR LINAC) platforms, and particle beam therapy^3–5^. With improvements in treatment precision and accuracy, stereotactic radiosurgery (SRS) and stereotactic body radiotherapy (SBRT) are increasingly utilized for the treatment of primary or metastatic disease, which has resulted in better tumour control and response rates associated with ablative doses of RT^6,7^. The basic therapeutic benefit of RT results from canonical DNA double-strand breaks from conventionally fractionated RT^8,9^. The better tumoricidal effects from SRS and SBRT using higher doses of RT per fraction (> 8–10 Gy) are believed to be linked to the activation of acid sphingomyelinase enzyme (ASMase) in the endothelial membranes leading to ceramide generation resulting in endothelial cell apoptosis^10–12^. Although higher-dose equivalents of radiation improve tumour control probability, a limiting factor is radiation dose to adjacent normal tissue structures despite the use of best conformal techniques. The search for ideal radiosensitizers (selectively affecting tumour cells) continues with several agents tried in the past, including various chemotherapeutic drugs, hypoxic modifiers, cell cycle modifiers, or physical agents like hyperthermia with variable degrees of success^13,14^. The use of current radiosensitizers are often limited by the increased normal tissue toxicities as the majority are administered systemically, or technical challenges in targeting entire tumor volume for complex deep-seated tumors. There are no chemical radiosensitizers investigated to date over the past 40-years, that have found widespread or daily usage in clinical radiation oncology.

Ultrasound (US) has a well established diagnostic role in contemporary medicine^15^. Therapeutic applications of US include a diverse spectrum of medical conditions like ablation of uterine fibroids, extracorporeal lithotripsy for renal stones, cataract surgery (phacoemulsification), intravascular thrombolysis, and surgical procedures (tissue dissection and vessel sealing)^16^. Microbubbles (MB) are used as US contrast agents and are composed of a gaseous core stabilized by a thin shell of biocompatible material like protein, lipid, or polymer. The diameters of MB range between 1 and 10 μm (typically 2–4 μm), allowing them to pass into tumour vessels through the systemic circulation with a typical half-life of 5–10 min. Acoustic stimulation of MB using ultrasound with an appropriate frequency and power can lead to their stable or inertial cavitation with the formation of local microjets and shockwaves, which can permeabilize adjacent endothelial cells. Applications of US-induced MB stimulation have demonstrated promise in targeted drug delivery, gene transfer, treatment of intravascular thrombi, or the transient opening of the blood–brain barrier^17,18^. In preclinical studies, including mice and rabbits, we have demonstrated the potent radioenhancing effect of FUS-MB therapy prior to RT, with tumour death increasing to 10–20 fold^19–21^. The ASMase pathway activated with higher ablative doses of radiation leading to apoptosis acts synergistically with other conventional ways of radiation-related cell death like mitotic death leading to additive benefits or tumoricidal effects tentatively improving tumor control probability. The importance of the ASMase pathway was first determined by Garcia-Barros et al. for large doses of radiotherapy^22^ and the role of that pathway in FUS-MB treatments was identified by Czarnota et al.^19^ and further elaborated by El Kaffas et al.^20^. The reason for this radioenhancing effect is the activation of ASMase and subsequent ceramide production leading to the activation of apoptotic pathways and endothelial cell death. In preclinical animal studies, the radiosensitization effect of FUS-MB has been demonstrated for both single fraction and fractionated radiation schedules. In the current human study, FUS-MB was used concurrently with standard fractionated radiation doses prescribed by radiation oncologists, typically delivered in 5–10 fractions. A number of preclinical studies involving over 3000 animals in total have now been conducted with recent work investigating the genetic pathways involved in vivo^20,23^. This leads to accelerated tumour cell death, as seen with otherwise ablative doses of RT. Focussed ultrasound (FUS) can be targeted conformally to the tumour, avoiding surrounding normal tissue, resulting in selective stimulation of MB with a desired target volume. Furthermore, FUS platforms can be integrated with an MRI platform for guiding treatment delivery with advanced imaging in real-time, improving treatment accuracy and precision.

The current study is the first human study to study the effects of MRI-guided FUS-MB therapy and external beam RT to demonstrate the feasibility and safety of this combined modality treatment, and to investigate the efficacy of the FUS-MB with RT. The first clinical trial of FUS-MB treatment for radiosensitization was started in patients with breast cancer due to the presence of gross disease in the breast or chest wall, which is technically easier to be targeted with this new modality. Contemporaneously, separate trials have been initiated and currently undergoing in head neck malignancies and melanoma. In the phase 1 study reported here, 9 tumours from 8 patients with breast carcinoma, specifically with gross unresected disease involving the breast or chest wall, were treated using FUS-MB and RT, showing promising antitumoural effects.

## Results

### Patient and tumour characteristics

Eight (8) women with a histological diagnosis of primary breast malignancy were treated with FUS-MB for 9 tumours (1 patient had bilateral breast disease) in the study. One patient (a ninth) was removed from the analysis as they did not present for follow-up. Individualized patient tumour characteristics, RT details, and outcomes are summarized in Table 1. Median patient age was 59 years, with 8 patients having invasive ductal carcinoma, and all 8 patients having simultaneous metastatic disease during FUS-MB. Hormone receptor-positive status was seen in 5, human epidermal growth factor receptor 2 (Her2) in 2, triple-negative in 3. Additional patient and treatment details are presented in Table 2.

**Table 1:** Tumour-wise treatment characteristics and outcomes.

Tumour number	Site	RT dose	Tumour/replacement fibrosis size at various intervals (mm)					Clinical outcomes		
			Pre RT	Post RT 1 week	Post RT 1 month	Post RT 3 months	Post RT 1 year	Toxicity (dermatitis)	Follow-up 3 months	Follow-up 12 months
1	Left retroareolar mass	40 Gy**/**10#	59 × 48 × 27	57 × 42 × 29	48 × 27 × 16	CR	CR	Grade 1	No tumour (complete response)	No tumour (complete response)
2L	Right breast lump with skin involvement	30 Gy/5#	24 × 24 × 16	26 × 21 × 12	24 × 21 × 12	15 × 12 × 15 (RF)	10 × 6 × 9 (RF)	Grade 1	No tumour (replacement fibrosis)	No tumour (replacement fibrosis)
2R	Left breast lump	30 Gy/5#	23 × 23 × 27	21 × 27 × 25	21 × 23 × 24	22 × 18 × 17 (RF)	15 × 12 × 12 (RF)	Grade 1	No tumour (replacement fibrosis)	No tumour (replacement fibrosis)
3	Right chest wall lump	30 Gy/5#	23 × 15 × 13	19 × 22 × 17	17 × 13 × 14	CR	CR	Grade 2	No tumour (complete response)	No tumour (complete response)
4	Right breast lump	30 Gy/10#	53 × 42 × 50	43 × 39 × 25	34 × 28 × 16	11 × 10 × 16 (RF)	CR	Grade 1	No tumour (replacement fibrosis)	No tumour (complete response)
5	Left breast lump	20 Gy**/**5#	20 × 28 × 38	17 × 20 × 17	16 × 14 × 15	14 × 13 × 14 (RF)	CR	Grade 1	No tumour (replacement fibrosis)	No tumour (complete response)
6	Left retroareolar lump	20 Gy/5#	37 × 27 × 26	36 × 32 × 23	35 × 24 × 27	30 × 26 × 25 (RF)	28 × 20 × 25 (RF)	Grade 1	Increase in left breast inflammatory area for re-irradiation surrounding treated region	No tumour (replacement fibrosis)
7	Left breast skin nodules	40 Gy**/**10#	62 × 45 × 39	30 × 35 × 31	23 × 24 × 18	CR	NA	Grade 1	No tumour (complete response)	No tumour at 9 months, died of metastatic disease at 9 months
8	Left chest wall lump with skin involvement adherent to chest wall	20 Gy**/**5#	88 × 65 × 68	63 × 102 × 39	61 × 63 × 28	NA	NA	Grade 1	Died of metastatic disease at 3 months	NA

*RT* radiotherapy, *CR* complete response, *RF* replacement fribrosis.

**Table 2:** Patient characteristics and treatment details.

Tumour number	Age (years)	Metastatic disease	Molecular status	Prior surgery (breast)	Prior RT to breast	Systemic therapy before three months of MR	Targeted tumour volume (MRgFUS-MB)	RT volume	Number of cells	FUS power	Systemic therapy after MRgFUS-MB
1	72	Bone, liver	TNBC	BCS	None	None	24 cc	Partial breast	3	4 Watts	Capecitabine
2L	75	Pleural effusion	ER + PR + HER2-	BCS	26 years back (details NA)	Palbociclib, Letrozole	5 cc	Partial breast	3	7 Watts	Palbociclib, Letrozole
2R	75	Pleural effusion	ER + PR + HER2-	BCS	26 years back (details NA)	Palbociclib, Letrozole	5 cc	Partial breast	3	4 Watts	Palbociclib, Letrozole
3	59	Neck nodes, bone, liver	ER-PR-HER2 +	Mastectomy	None	Trastuzumab emtansine, Capecitabine	19 cc	Partial breast	4	6 Watts	Trastuzumab emtansine, Capecitabine
4	44	None	TNBC	BCS	50 Gy/25fractions (2 years back)	None	36 cc	Whole breast	6	4 Watts	Capecitabine
5	59	Neck nodes, bone, lung	ER + PR + HER2-	None	None	None	10 cc	Partial breast	3	8 Watts	Fulvestrant, capecitabine
6	76	Lung	ER + PR + HER2-	BCS	50 Gy/25 fractions (35 years back)	Capecitabine	32 cc	Partial breast	5	5 Watts	Tamoxifen, Olaparib
7	57	Lungs, pleural effusion, retroperitoneal nodes	ER + PR-HER2 +	None	None	Trastuzumab	29 cc (skin nodules over medial aspect)	Whole breast	7	4 Watts	Nab-paclitaxel, neratinib, capecitabine, trastuzumab
8	61	Lung	TNBC	Mastectomy	50 Gy/25 fractions (1 year back)	Gemcitabine, Carboplatin, Atezolizumab	76 cc (skin nodules over the medial aspect of scar and chest wall)	Whole breast	8	4 Watts	Gemcitabine, Carboplatin, Atezolizumab

### Treatment details

Prior to recurrence development and study treatment, 6 patients had originally undergone breast surgery (mastectomy in 2, breast-conserving surgery in 4), and RT to the breast or chest wall had been done in the past in 4 patients. No specific radiation dose fractionation schedules were used for the study. The radiation oncologists had decided on the doses as per their standard practice and preference without the influence of the current study. Typically, the standard radiation doses in the palliative setting in our institute include 5 fractions (20–30 Gy) or 10 fractions (30–40 Gy). Along with FUS-MB treatment, whole-breast RT was carried out in 4 patients, while focal RT to the tumour with margins was carried out for 4 tumours. Radiation doses included 20 Gy in 5 fractions (3 tumour sites), 30 Gy in 5 fractions (3 tumour sites), 30 Gy in 10 fractions (1 tumour site), and 40 Gy in 10 fractions (2 tumour sites). For all patients, FUS-MB was delivered before the 1st and 5th fraction of RT. Details regarding FUS-MB and systemic therapy are presented in Tables 1 and 2. Of 8 patients included in the analysis, 5 had received systemic therapy before FUS-MB treatment. After completion of radiation, systemic therapy was given to all patients, with 3 patients continuing the same line of treatment until the last follow-up, and the remaining 5 receiving multiple lines of systemic therapy (including hormonal therapy, cyclin-dependant kinase inhibitor, chemotherapy, and targeted therapy). The median target volume with FUS-MB was 24 cm^3^ (range 5–76 cm^3^), with a median number of FUS cells being 4 (range 3–8). The median treatment time for the 1^st^ FUS-MB treatment (including post-treatment CE-MRI) was 77 min (range 49–112 min), and for the second treatment, the median time was 71 min (range 27–122 min). All the patients were able to complete FUS-MB treatments.

### Safety

**Figure 1:**
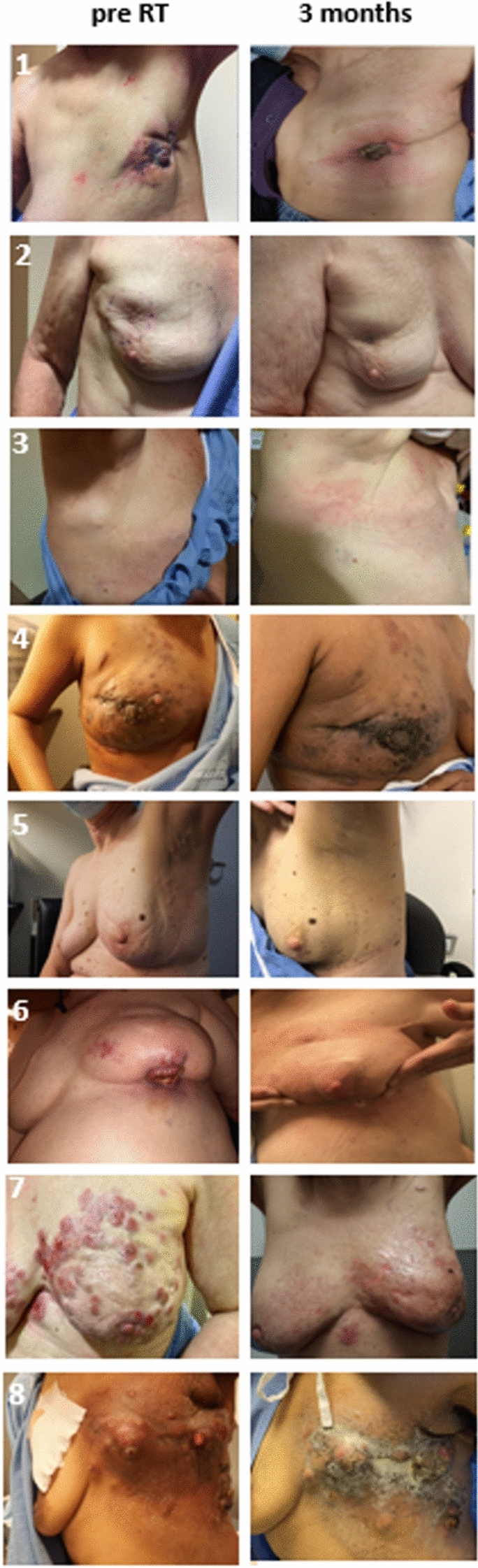
Clinical photographs demonstrating clinical tumour conditions before treatment and after 3 months of radiation for individual patients. Note for patient 8, the images are shown at 1-month post-treatment since follow-up at 3 months was not available.

None of the patients had any systemic complications or allergic reactions to the FUS-MB treatment. The degrees of acute toxicity (within 3 months of radiation completion) observed were grade 1 radiation dermatitis at 8 tumour sites, and grade 2 dermatitis in 1. For 6 evaluable patients at 12 months, all dermatitis had resolved. No RT late effect (e.g., RT-induced skin fibrosis, rib fractures, or brachial plexopathy) was seen. Figure 1 presents the clinical photographs for patients before and at 3 months after FUS-MB and RT treatment, showing the skin condition and any visible tumour to inspection (when applicable for patients with tumours having skin involvement).

### Treatment response

**Figure 2:**
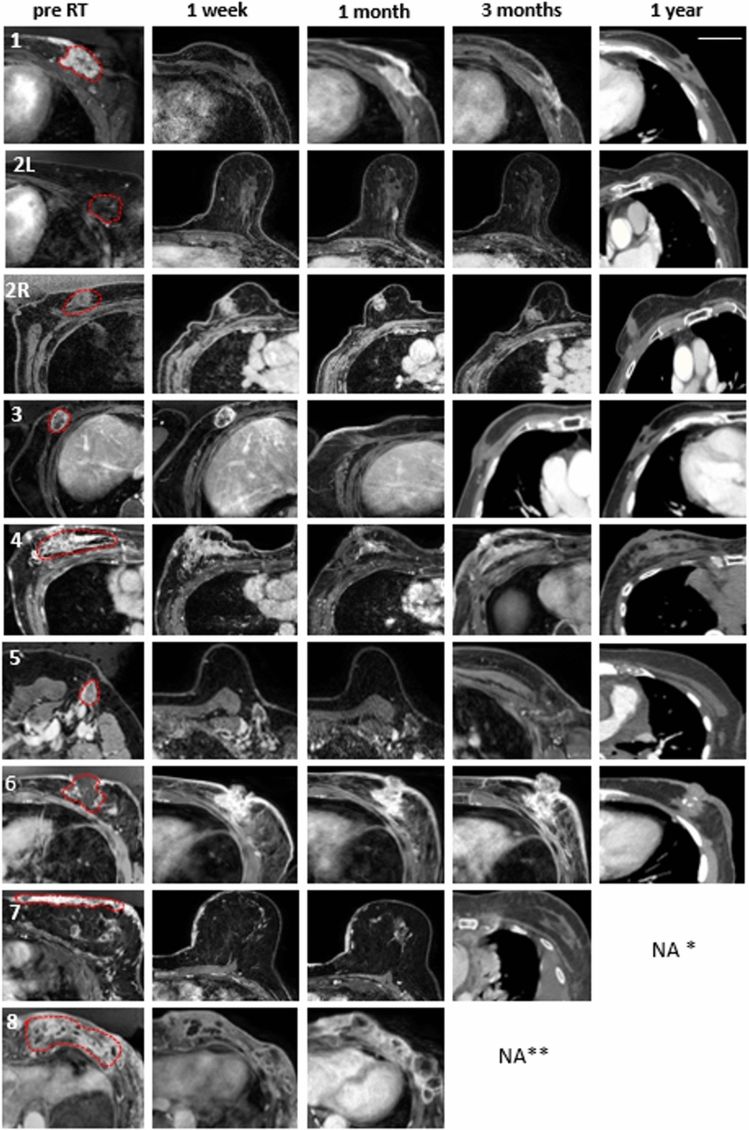
Individual tumour imaging before and after treatment The panel on the left represents pre-radiation target volume indicated, while the subsequent images show radiological outcomes with T1-weighted contrast-enhanced MRI at 1 week, 1 month, 3 months, and CT image at 1 year after treatment. *Patient 7 died of metastatic disease at 9 months (1-year follow-up not available), **Patent 8 died of metastatic disease at 3 months (3 months and 1-year follow-up not available). The scale on the top right figure represents an approximate size of 5 cm.

**Figure 3:**
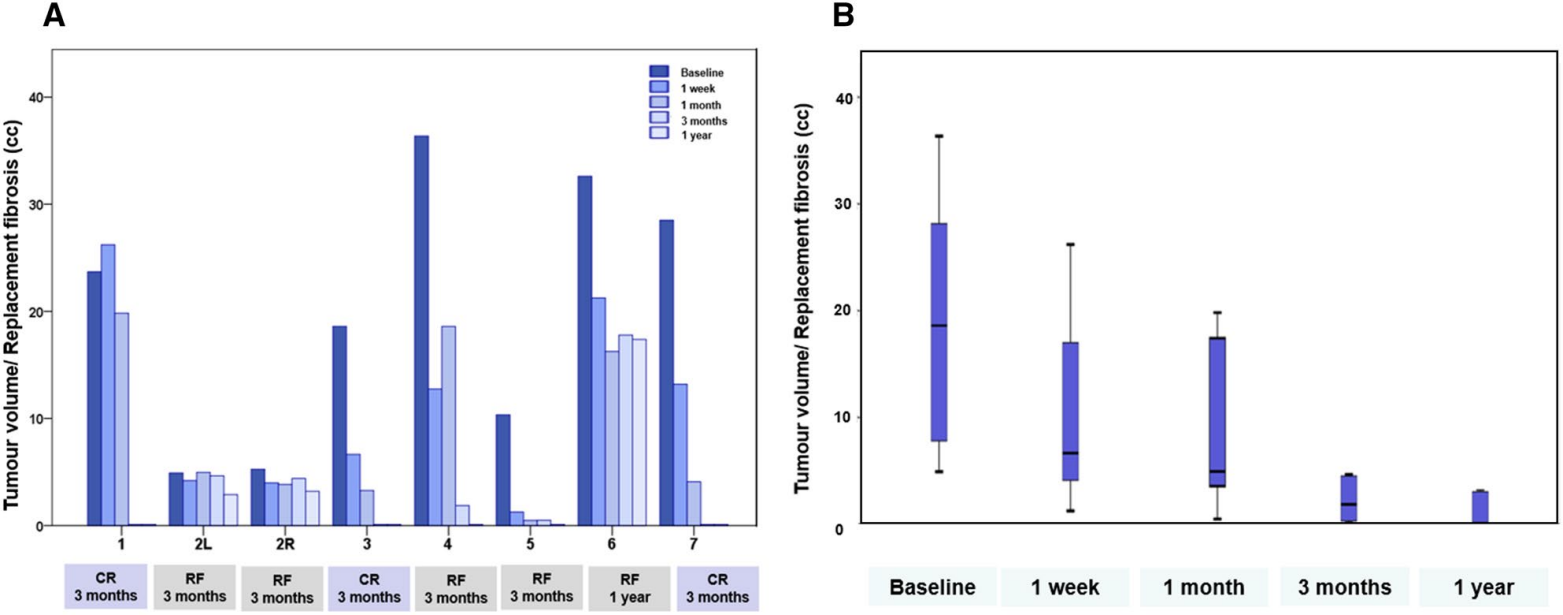
(**A**) Individual tumour volumetric responses at different times for patients with at least 3 months of follow-up. (**B**) The volumetric response for all patients having 1 year of follow-up. *CR* complete response, *RF* replacement fibrosis.

Of the 8 patients treated in the study, 6 patients (7 tumours) had follow-up at 12 months post FUS-MB treatment. One other patient declined any further visits due to mental illness after FUS-MB and was non-evaluable (excluded from analysis and summary), while 2 patients died of progressive distant metastatic disease unrelated to the site of treatment at 3 and 9 months from treatment. Figure 2 presents the radiological response for individual patient tumours before treatment and at different follow-up times after treatment completion. Complete response (CR) following FUS-MB and RT, with the absence of enhancing radiological and clinical disease, was observed in 3 of 8 tumours at 3 months of follow-up. A residual non-enhancing fibrotic area was observed in the remaining 4 patients at 3 months (one patient died of distant metastatic disease) interpreted as replacement fibrosis (RF) due to treatment. All evaluable 7 patients at 12 months had complete response (n = 4) or RF (n = 3), without evidence of any active or recurrent disease in the treated site. In the one patient who died before 3 months, the treated tumour was stable in appearance at 1 month. The other patient who died before 12 months had a CR at 9 months when she succumbed to metastatic disease (brain metastases). Individual tumour response with measurable volumetric radiological disease or fibrotic areas in patients with a follow-up of at least 3 months is detailed in Table 2 and shown in Fig. 3A. The combined volumetric radiological response for 7 tumours with 12 months follow-up is presented in Fig. 3B.

## Discussion

Higher doses of radiation can potentially improve tumoricidal effects, but with increased concerns for normal tissue complications, Dose increases mandate optimizing therapeutic ratios in order to balance cure with an acceptable probability of adverse effects^24^. The use of radiosensitizers or other therapies that can selectively enhance the effect of RT in target tissue without affecting the adjacent normal tissues can increase the therapeutic ratio, improving tumour control probability without causing additional risk of radiation-related toxicities^13,25^. With promising results from preclinical studies using MRI-guided FUS-MB as a radioenhancer which targets tumour vasculature through the ASMase pathway and ceramide-related endothelial cell apoptosis, we present the first human application of FUS-MB combined with RT in breast malignancies.

The primary driving principle of FUS-MB as a radiosensitizer is the acoustic stimulation of MB using low mechanical-index ultrasound. Stable and inertial MB-cavitation leads to stress on endothelial cells as well as rupture of MB with the formation of microjets and acoustico-mechanical effects on endothelial cells. Subsequent activation of the ASMase-ceramide pathway in endothelial cells leads to apoptotic cell death. In this mechanism, destruction of the tumour vasculature leads to anoxic death of tumour cells—beyond the premises of hypoxia. It is well known that hypoxia increases radioresistance, reducing the therapeutic effect of RT. Here effects are linked rather to ischemic death of tumour cells. In preclinical experiments on cancer xenograft-bearing mice treated with multiple combinations with and without MB and different doses of single-fraction RT demonstrated supra-additive effects with combined modality, resulting in a higher proportion of apoptotic cell death^19,26^. In preclinical research, combined treatments in xenografts led to increased cell death within 24 h after combining treatments with 2 or 8 Gy single fractions of radiotherapy, respectively. When a single 2 Gy radiation was used, cell death with apoptosis or necrosis was seen in 4 ± 2% (mean ± SE), while the use of ultrasound-microbubble treatment without radiation led to cell death in 10 ± 4%. The fraction of cell death in the irradiated region was remarkably increased when ultrasound-activated microbubble treatment was done, both for low (40 ± 8%) and high ((44 ± 13%)) microbubble concentrations. Similarly, 8 Gy of radiation with microbubble activation led to a higher proportion of cell death (70 ± 8%). To summarize, ultrasound stimulation of microbubbles before radiation resulted in a supra-additive effect of the two modalities^19^. Multiple other studies with other tumour lines have since been conducted^26^. For the patients treated in the study with a combination of FUS-MB and radiation, both pathways were supposedly activated, including radiation-induced DNA damage and FUS-MB induced ASMase activation leading to apoptosis. Therefore, the high rates of disease resolution (complete response) can be postulated by the combined effect of both mechanisms acting in an additive way leading to enhanced cell death in the tumour.

In the original work, interestingly, the majority of the lethal effect was due to the MB treatment, accounting for approximately 70% of the observed effect^19^. Similar results were observed in fibrosarcoma xenografted mice, and the biological basis of the ASMase pathway in FUS-MB treatment was established since ASMase knockout mice and groups treated with sphingosine-1-phosphate (ASMase-ceramide pathway inhibitor) failed to demonstrate radioenhanced effects^20^. Tumour perfusion with FUS-MB was studied using 3D Doppler ultrasound, which demonstrated a decrease in tumour perfusion by up to 47% by 3 h with FUS-MB and RT treatment, which peaked at 24 h. The radiosensitization effect of FUS-MB with fractionated radiotherapy was demonstrated in rabbits with prostate tumour xenografts, which had shown a significant reduction in tumour size after 3 weeks of combined therapy and superior animal survivability^21^. The added advantage of using FUS-MB as compared to pharmacological agents is the physical conformity of FUS with a penumbra of < 1 mm and accurate delivery made possible by MRI guidance resulting in radioenhancement only in the desired tumour volume and at the site of FUS focus.

Radiation is commonly used as an adjuvant treatment modality in breast malignancies following surgery^27,28^. In selected instances, RT is used to treat gross disease in the breast or chest wall when a patient is not eligible for surgery, has an inoperable disease, or breast cancer is being treated in a recurrent setting for local control or symptomatic palliation^29,30^. Although locoregional RT can result in some degree of tumour control, complete response rates to definitive RT for gross macroscopic breast disease are often unsatisfactory. In the current study, in all seven evaluable tumours at 12 months, the study here demonstrated 100% CR rates. In many patients, a residual fibrotic area was evident upon physical examination, with an absence of contrast enhancement on MRI or CT indicating the absence of gross disease. None of the patients in the study at any time point had demonstrated disease recurrence in the site treated with FUS-MB and RT. Although the sample size here was small, the increased RT response rates are promising at one year of follow-up, indicating a potent radioenhancing effect of FUS-MB with complete response or replacement of disease with complete fibrotic changes.

Recent advancements in therapeutic ultrasound delivery platforms have enabled accurate delivery of US and, although here tied to MR imaging, can be coupled to other imaging modalities^16^. The technology can be used to enhance radiation effects making treatments more efficacious. Another critical application of using combined FUS-MB and RT treatment will be in the reirradiation setting, where normal tissue complications can be potentially minimized by acoustic stimulation of microbubbles in the tumour target region along with lower doses of RT. Like RT, which can be conformed to tumour geometry in three-dimensions focussed ultrasound can be delivered with an even tighter penumbra of microns. Areas in the ultrasound near-field and far-field (out-of-focus) have no appreciable effect on microbubble cavitation, similarly permitting depth-based conformality in treatment.

A similar FUS-MB approach has been used with other vasculature-targeting methods involving radiation, such as TARE (transarterial radioembolization) with technetium-99 macroaggregated albumin. Preliminary efficacy results from that study indicated a greater prevalence of tumor response (14 of 15 [93%; 95% CI 68, 100] versus five of 10 [50%; 95% CI 19, 81]; P = 0.02) in participants who underwent both US-triggered MB destruction and TARE (P = 0.02) compared to TARE alone^31^.

For future consideration, the microbubbles can be engineered to contain and deliver a wide range of chemotherapeutic or targeted therapy applicable for specific types of malignancies, being investigated in preclinical and early clinical trials. This can trigger drug release selectively within the tumour only using FUS, increasing drug concentration in the target tissue and avoiding systemic complications. FUS has also shown promises in the transient opening of the blood–brain barrier (BBB), resulting in better drug delivery for different intracranial pathologies and brain tumours and combinations with radiotherapy^32^.

At present, FUS devices for treatment are mostly either handheld adapted diagnostic systems or MRI-guided technology. One limitation of the approach here which used a HIFU-device reprogrammed for low-power non-temperature elevating ultrasound as a step and shoot approach, which despite broadening target treatment cells still had to be used. Newer versions of FUS equipment will include a larger array of treatment cells able to encompass larger tumor volumes, and treatment times also can be reduced in that manner^33^. Also, it will enable the targeting of deeper-seated tumors with complex geometric shapes, and volumes can be targeted with a higher degree of conformality.

The work here was carried out on breast cancer patients with gross macroscopic disease. Most had wide-spread metastases elsewhere, on follow-up amongst all patients alive at 12–16 months no recurrence was evident at the treated disease site. The implications of factors like molecular status and systemic agents that can have possible implications on tumour response were not addressed in the study here and will be accounted for as stratification factors in future work. With a small number of patients included in the phase 1 study, the safety of the combined treatment modality has been established along with potential efficacy to improve treatment response. A subsequent randomized study is planned to include a control group and larger number of patients for better interpretation of efficacy of FUS-MB treatment.

## Conclusion

The work here represents the first use of FUS-MB with external beam RT on tumour treatments eliciting complete and durable responses in patients. Replacement fibrosis of the tumour occupying area was common in responding patients. The safety of combined modality treatment was demonstrated along with promising results showing potent anti-tumoural effects. The study opens up a new frontier of using FUS-MB as an effective and safe radioenhancer and can potentially change existing paradigms of radiation oncology practice improving therapeutic treatment ratios.

## Methods

### Study design and participants

This prospective phase 1 interventional study was conducted at Sunnybrook Health Sciences Centre, Toronto, as an investigator-initiated study approved by the Sunnybrook Health Sciences Centre research ethics committee and registered with clinicaltrials.gov (identifier NCT04431674) on 16.06.2020. The study was conducted following good clinical practice (GCP) and according to the Helsinki declarations. Patients with a diagnosis of breast malignancy having primary or recurrent disease in the breast or chest wall requiring RT as decided by a multidisciplinary team of medical, surgical, and radiation oncologists were eligible for the study. Study inclusion criteria involved age ≥ 18 years with normal coagulation profile, normal liver and renal function tests (a prerequisite for MB treatment), and weight < 140 kg (a requirement for being able to be scanned on the MRI machine). Exclusion criteria included pregnant or lactating women, unable to undergo contrast-enhanced MRI, presence of breast or other metallic implants, index lesions with ulceration or bleeding, presence of fibrotic scar in the US beam path, history of allergy to microbubbles, history of cardiac disease, uncontrolled hypertension (diastolic BP > 100 mm Hg), history of coagulopathy, use of anticoagulants, estimated glomerular filtration rate < 30 ml/min, and Eastern Cooperative Oncology Group (ECOG) performance ≥ 3. All study participants signed a written consent form before study participation.

### Procedures

Decisions regarding RT target volumes treated (tumour or whole breast) and radiation dose prescribed was taken by the responsible radiation oncologist and were not influenced by participation in the current study. As a part of the study protocol, each patient underwent gadolinium-based (Gadovist, 1 ml/kg, 1 mmol/ml) contrast-enhanced 3T magnetic resonance imaging (CE MRI), the information of which was available for US and RT planning. Patients were positioned supine with arms placed overhead, and non-contrast computed tomography (CT) scans were additionally acquired for RT planning. Radiation treatments were delivered on a standard linear accelerator equipped with image guidance using 3-dimension conformal or intensity-modulated radiotherapy therapy. Before treatment, T1 and T2-weighted MRI was undertaken for all patients, which was used in target volume delineation for radiation and also for preplanning of FUS-MB treatment. Disease, as appreciated on MRI and clinically, was targeted based on discussion with the most responsible radiation oncologists and radiologists to include the same target defined for radiotherapy. Once the target area was defined, the path of the least distance from the surface to the tumor was planned. Accordingly, the treatment cells for FUS-MB were placed with adjacency in order to encompass the entire volume identified. On the day of treatment, the final decision with minor adjustments of treatment cells was made based on the MRI undertaken in the treatment position. Definity microbubbles (Lantheus Medical Imaging, Billerica, MA, USA) consisting of perfluoro-propane within a lipid shell were used for the study. The FUS device was a Profound Medical Sonalleve device integrated into the couch of a 3T MRI (Philips, Achieva). FUS Parameters were customized to a frequency of 800 kHz with a peak negative pressure of 570 kPa based on experimental preclinical data. The power level at treatment depth was kept consistent by adjusting for depth-related attenuation using an attenuation co-efficient of 1 dB/MHz/cm^34,35^. A pulse sequence based on a 16-cycle tone burst over 50 ms was used. A delay time of 1950 ms was used before repeating the sequence over a 5–10 min period, for a total insonication time of 7500 ms. No heating is induced with these parameters. The device provides insonication to the selected area with high precision with a penumbra of 60 microns or less. Individual treatment cells were placed to cover the entire target volume, which was subsequently targeted individually in a phased manner by the FUS device. The individual cells were cylindrical with an approximate height of 2.8 cm (along the beam axis), and a diameter of 1 cm. Depending upon the depth of the tumour, an appropriate power was selected, accounting for attenuation factors to deliver the peak negative pressure in the cell centre. Cells were treated using a step and shoot approach.

The RT and FUS-MB treatments were carried out on an outpatient basis. For all patients included in the study, 5 or 10 fractions of RT were delivered. FUS-MB treatment was done on days 1 and 5 of RT. On the days of FUS-MB therapy, an antecubital intravenous cannula was inserted for injecting MB and MRI contrast. For FUS-MB treatments, patients were positioned prone with the target area placed over the US transducer table top region mounted on the MRI couch. To account for contour irregularity or tissue gap between the transducer and the target, ultrasound gel pads (Aquaflex; Parker, Hannover, Germany) measuring 4.0 cm (height) × 27.5 cm × 27.5 cm (length × width) were placed between the patient and ultrasound transducer table top. Before the actual treatment delivery, MRI scans with T1-weighted sequences were taken in order to ensure the absence of any air gaps (impeding ultrasound) and the feasibility of the target being covered by FUS. Any patient position adjustments were made as required. Once treatment compatibility was established, Definity MB (Lantheus Medical) were injected at 10 µL/kg, followed by 50 ml saline flush for each treatment cell, which was then insonified. Prior to using MB, they were removed from 4 °C storage, and the MB suspension was activated using a Vialmix device (Lantheus Medical Imaging, USA) using a 45-s cycle. The process was repeated for each treatment cell until treatment (MB and FUS) was completed for all the preplanned cells encompassing the entire target. At the end of FUS-MB treatment, a contrast-enhanced T1-weighted scan was performed after injecting 0.1 ml/kg of MRI contrast. The patient was then treated with RT within 60 min of FUS-MB exposure.

**Figure 4:**
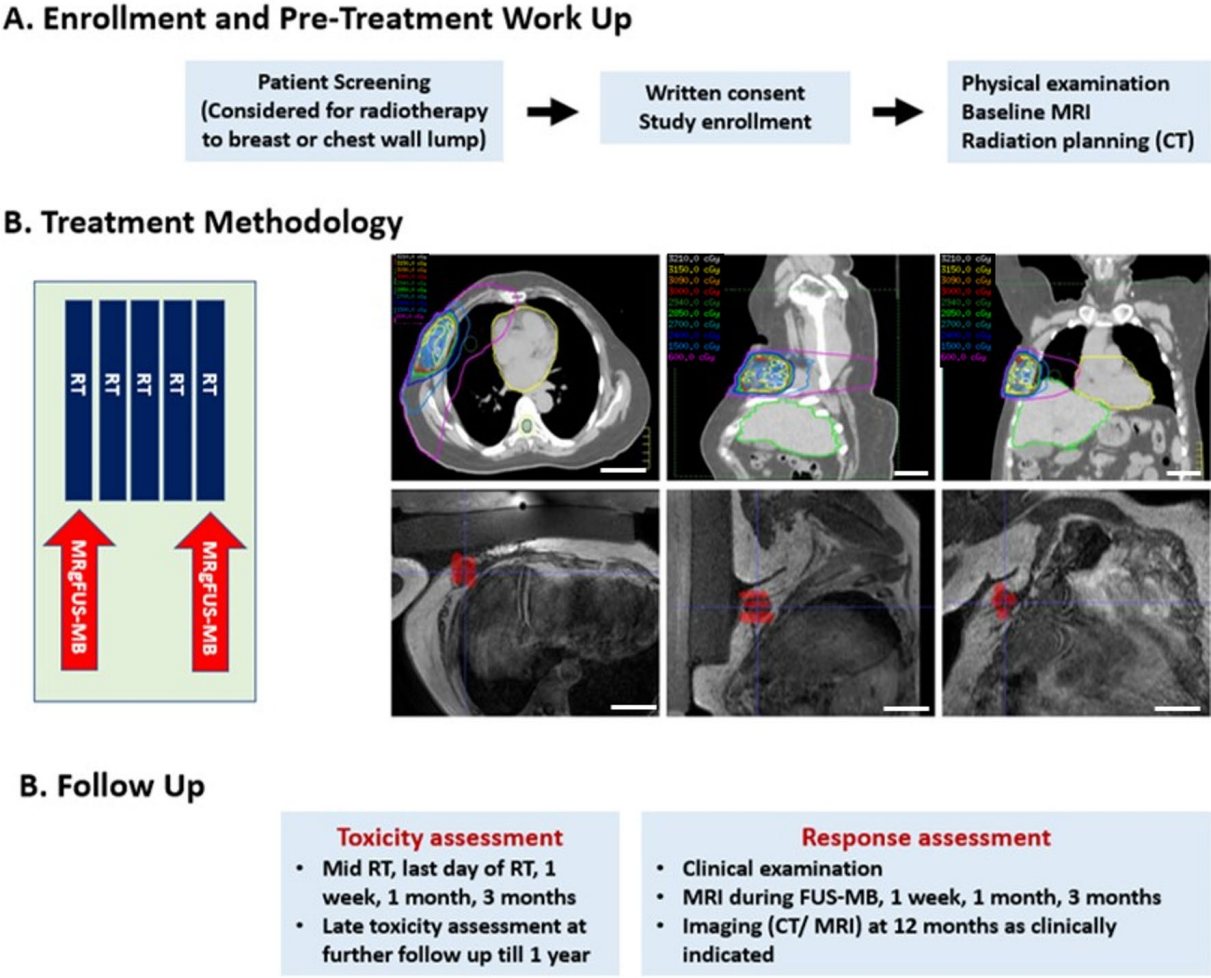
Study methodology, including follow-up and assessment protocols. Section B (methodology) shows the treatment methodology for a patient treated with FUS-MB before the first fraction of radiotherapy. The upper panel of the figures represents the axial, sagittal, and coronal computed tomography images of the radiation plan with radiation isodose lines (as shown in the upper left corner of the individual figures). The lower panel shows corresponding magnetic resonance images with cells (in red) for insonication. The scale on the lower right corner of individual figures represents an approximate size of 5 cm.

For patients on systemic therapy, RT treatment was scheduled during time off systemic therapy in order to minimize any potential interactions with the investigational therapy as would have been done for treatment with radiotherapy alone. Otherwise, all forms of systemic therapy (except hormonal therapy) were stopped 1 week before, during, and for after 1 week of completion of RT. After the FUS-MB and RT treatment, during follow-up, any decisions regarding systemic therapy were taken by the responsible medical oncologist, depending upon the patient's disease. The study methodology has been summarized in Fig. 4.

### Outcomes

Patient, disease, and treatment-related characteristics were collected from electronic medical records, patient charts and recorded prospectively. On the days of FUS-MB treatments, patients were monitored for 1–2 h after MB injection to ensure no associated complications developed (e.g. allergy, cardiovascular events). Safety data for each patient were assessed individually by the principal investigator, one study co-investigator, and one radiation oncologist independent of the study at 2 weeks following FUS-MB and RT treatment. Assessment for acute radiation toxicity was done using the Common Terminology Criteria for Adverse Events (CTCAE) version 4 at the middle of RT (for treatments > 1 week), on the last day of RT, and then at 1 month and 3 months after treatment completion. Patients were examined during each follow-up visit until 1 year after treatment for the assessment of any radiation-related late adverse events. Any adverse events were reported to the designated trial monitoring committee within the stipulated regulatory time.

For the assessment of treatment response, CE-MRI was carried out after 1 week, 1 month, and 3 months of study completion, along with clinical examination. Further imaging investigations, including CT or MRI, were performed as clinically indicated, decided by the treating oncologists without any influence of study participation. The disease targeted with FUS-MB and RT was assessed by dimension-based volumetric measurements from T1-weighted MR imaging. The presence of residual disease or treatment-induced fibrosis was decided based on clinical examination and radiology reports and compared with the pre-treatment imaging. Complete response (CR) was defined as the absence of residual disease on MRI T1-weighted images. Residual/replacement fibrosis (RF) was identified through non-uptake of MRI or CT contrast (baseline imaging demonstrating contrast enhancement). No biopsy or surgery was done in the study for pathological confirmation of response or fibrosis.

### Statistical analysis

Statistical analysis was performed using individual patient demographic, and disease characteristics and outcomes were presented using descriptive statistics as appropriate.

## Data Availability

Data are stored in an institutional repository and will be made available on request to the corresponding author following institutional ethics committee protocols.
